# Automated tracking and classification of the settlement behaviour of barnacle cyprids

**DOI:** 10.1098/rsif.2016.0957

**Published:** 2017-03-29

**Authors:** Ahmad Alsaab, Nick Aldred, Anthony S. Clare

**Affiliations:** School of Marine Science and Technology, Newcastle University, Newcastle upon Tyne NE1 7RU, UK

**Keywords:** barnacles, cyprid, settlement behaviour, tracking, classification

## Abstract

A focus on the development of nontoxic coatings to control marine biofouling has led to increasing interest in the settlement behaviour of fouling organisms. Barnacles pose a significant fouling challenge and accordingly the behaviour of their settlement-stage cypris larva (cyprid) has attracted much attention, yet remains poorly understood. Tracking technologies have been developed that quantify cyprid movement, but none have successfully automated data acquisition over the prolonged periods necessary to capture and identify the full repertoire of behaviours, from alighting on a surface to permanent attachment. Here we outline a new tracking system and a novel classification system for identifying and quantifying the exploratory behaviour of cyprids. The combined system enables, for the first time, tracking of multiple larvae, simultaneously, over long periods (hours), followed by automatic classification of typical cyprid behaviours into swimming, wide search, close search and inspection events. The system has been evaluated by comparing settlement behaviour in the light and dark (infrared illumination) and tracking one of a group of 25 cyprids from the water column to settlement over the course of 5 h. Having removed a significant technical barrier to progress in the field, it is anticipated that the system will accelerate our understanding of the process of surface selection and settlement by barnacles.

## 1. Introduction

Many sessile marine invertebrates have larval dispersal stages that actively select surfaces for attachment and growth to adulthood. In the context of human activity in the oceans, this process is referred to as marine biofouling—the unwanted accumulation of animals and plants on structures immersed in the sea. Biofouling has significant cost implications for maritime activities [1]. Heavy ship-hull fouling can reduce propulsion efficiency by as much as 86% [2]. The total financial burden of fouling prevention and amelioration is difficult to estimate, primarily because of fluctuations in bunker fuel price, but certainly runs into billions (USD) annually. A recent estimate for US Navy vessels put the cost for coating, cleaning and repairing the fleet owing to fouling accumulation at $180 million to $260 million (USD) per annum [3]. Biofouling, via the drag penalty it imposes, increases greenhouse gas emissions by global shipping [4], and it is the primary vector for translocation of non-native marine species around the world's oceans [5,6]. The means to control such fouling—biocidal hull coatings—rely primarily on toxic heavy metals for their antifouling efficacy [7,8] and retain a majority market share over non-biocidal alternatives. The latter, which usually function by minimizing the adhesion strength of attached fouling organisms, rather than repelling or killing them, have a higher initial cost to the ship owner and lack universal applicability. Biofouling and its control are therefore longstanding global problems impacting both economies and the environment.

**Figure 1.**
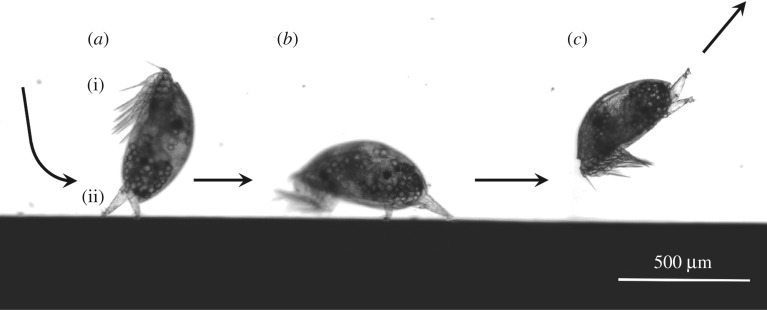
A cyprid (*Balanus amphitrite*) during typical surface exploration behaviour, viewed from the side for illustrative purposes: (*a*) initial contact with the surface and temporary adhesion, (i) thoracic swimming appendages, (ii) the paired antennules used for temporary adhesion during exploration of surfaces. (*b*) Typical posture adopted by a cyprid during wide search, close search and inspection behaviours. (*c*) Detachment from the surface and returning to the water column.

Of the invertebrate taxa that are found routinely in fouling consortia, barnacles are among the most widespread and significant in terms of their economic impact [9]. Further, the settling larval stage of barnacles (cyprid) displays complex pre-settlement behaviour that enables surface selectivity [10]. The cyprid attaches and explores surfaces by means of a temporary adhesion mechanism [11], involving two frontal antennules and their associated adhesion apparatus—a pair of ‘hairy’ attachment discs and a viscous temporary adhesive secretion [12]. In this way, cyprids are able to walk in a bipedal fashion across immersed surfaces, choosing whether to explore further or to leave and investigate alternative substrata (figure 1). Substrate choice is a key determinant of future survival in barnacles and, consequently, the adoption of barnacles as model species in marine fouling studies has been motivated as much by fundamental curiosity into the larval decision-making process as it has by applied interest in ship efficiency [13].

Cyprids select or reject surfaces they encounter on the basis of the biological, chemical and physical characteristics of the surfaces and of the surrounding environment [14,15]. While some of the responses to these cues and signals are understood for particular species, little is known of the endogenous mechanisms that process the sensory information and lead to acceptance (permanent attachment) or rejection of a surface. Fundamental questions that remain to be resolved in relation to surface selection include: What are the functions of the sensory setae of the ambulatory cyprid antennules [16–18]? What is the role of the compound eyes [19] which are only present only in the cyprid stage [20]? To what degree do previous encounters influence the decision to settle? The answers to such questions may find direct application in the development of surfaces that are rejected and thus remain free of barnacle fouling.

Historically, the pre-settlement behaviour of cyprids has been classified under four broad headings: swimming, wide search behaviour, close search behaviour and inspection [10,21]. These classifications refer to a generally accepted model of cyprid exploratory behaviour that postulates initial exploratory coverage of a large surface area with few turns (wide search), followed by an increasing number of sharper turns if the surface remains acceptable (close search). This process of increasingly refined surface exploration is, it is believed, followed by a period of inspection prior to permanent adhesion. During inspection, the cyprid remains in one location ‘probing’ the area immediately surrounding the attachment point with its antennules and associated sensory apparatus, comprising several setae. While these behaviours certainly occur and are easily observable in the laboratory, how accurate, broadly applicable (Crisp's observations were limited to one barnacle species, namely *Semibalanus balanoides* [10]) and predictably sequential the Crisp model [10] as a whole might be remain unknown. The uncertainty stems from technical limitations in behavioural experiments using cyprids. Without the means to reliably acquire large quantities of non-subjective data and determine a rigorous conceptual framework for cyprid behaviour as a baseline, wide use of behavioural analysis for practical surface evaluation remains premature.

Development of a system to achieve the above is not a trivial exercise. While it would seem obvious for investigators of cyprid behaviour to draw inspiration from elegant motion-tracking methods used to study marine zooplankton [22], published accounts focus invariably on swimming. This is the least challenging behaviour to identify and although crucial to the ability of the cyprid to attach in flow [23], it is also the least interesting behaviour with respect to surface selectivity. Investigators of cyprid behaviour have reported a diversity of obstacles to progress in their efforts to develop cyprid-specific techniques, although these usually converge on the inability of available methods to distinguish between swimming and the three above-mentioned exploratory behaviours. This superficially tractable situation belies a complex set of interconnected technical (e.g. image resolution, frame rate, lighting, cyprid shape, size-to-speed ratio, classification of behaviours) and biological (e.g. sporadic, variable behaviour, protracted sequence leading to settlement, lack of ecological context) hurdles to be overcome. Quantifying the surface-specific behaviour of cyprids is significantly more complex than tracking the movement of an object in two or three dimensions, not least, because swimming behaviour (which may account for more than 90% of experimental data in a cyprid-tracking experiment) must first be partitioned from the dataset before identification of the subtler exploratory movements can begin.

The most important and most challenging aspect of quantifying and comparing cyprid behaviour between treatments is that any comparative measure of behaviour must eliminate the subjective judgement of the experimenter and, thus, be entirely automated. Attempts to achieve this began with studies relying on commercial software, such as Noldus EthoVision v. 3.0/3.1 [24–26]. While these commercial software packages and their modern equivalents are adequate for describing the overall movement of solitary cyprids in a small arena, they do not allow for distinction between swimming and exploration behaviours. The development of three-dimensional stereoscopic-tracking systems provided certainty regarding the vertical position of cyprids in the water column; however these systems are complex to implement and do not, *per se*, increase the ability of the experimenter to differentiate between exploratory and non-exploratory behaviour. The introduction of a novel method to track multiple cyprids simultaneously [27] was another notable advance, but the research has remained restricted by manual classification of cyprid behaviours [28]. Similarly, although Pradhan *et al*. [29] developed an automatic classification method to detect the wide and close search behaviour of a single cyprid, their imaging system was mounted horizontally and wide search behaviour was simply defined as when the cyprid moved approximately horizontally to the surface (a behaviour that often occurs during swimming). Close search was then defined when the cyprid positioned its body towards vertical with respect to the surface. Again there are significant practical drawbacks with this approach, such as movement towards and away from the camera remaining undetectable. More recently, Maleschlijski *et al*. [30] combined a stereoscopic system for tracking cyprid movement with a novel method for visualizing cyprid–surface interactions based upon imaging surface plasmon resonance. In principle, this combination of techniques should allow correlation of macroscale movement in the bulk liquid to microscale exploratory interactions with a surface. In practice, the combination of methods to achieve this requires significant technical knowledge, and data collection is both spatially and temporally limited. While the surface plasmon resonance-based technique has significant potential to answer questions regarding the temporary adhesive interactions of cyprids with surfaces during exploration, it is not appropriate for investigating global cyprid behaviour and the processes that lead to settlement.

All methods reported so far share an additional common problem—the difficulty of dealing with cyprids that overlap in the field of view. This has led to experimental approaches that either rely on data from individual replicate larvae [31] (a time-consuming approach with a low data yield) or use of multiple larvae simultaneously with the acceptance that confidence in the data will be limited to short stretches in between overlapping events, preventing long-term assessment of the behaviour of individual cyprids [28].

In this paper, we describe the development of the first software designed specifically to track and analyse cyprid exploratory behaviour. Using only a single camera, the tracking system is capable of reliably tracking multiple cyprids simultaneously for long periods. The system thus enables the user to conduct long-term experiments (hours) with minimal intervention and opens the door to the in-depth study of behaviours leading to settlement in barnacle larvae. Further, we describe a classification system that was designed concomitantly with the tracking system to automatically and reliably partition exploratory behaviour into wide search, close search and inspection events. Examples are given in the form of two lighting conditions that invoke significantly different proportions of these behaviours and a representative track of a cyprid during its transition from a free-swimming larva to settlement and metamorphosis.

## 2. Development of a cyprid-specific tracking system

### 2.1. Materials

**Figure 2.**
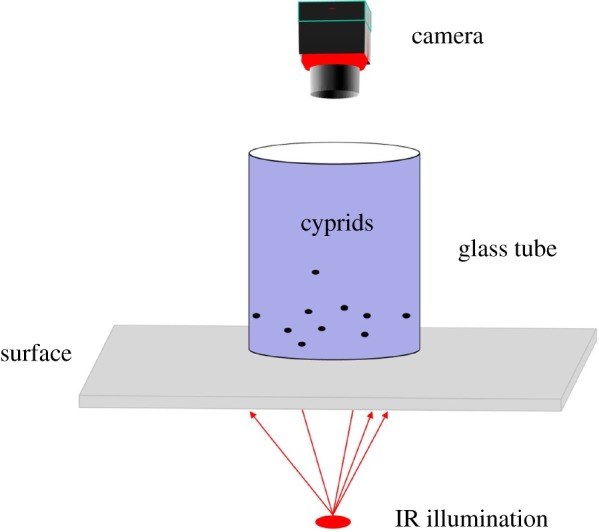
The image acquisition system.

The simple image acquisition system consisted of a PC and a Basler scS1300-32gm monochrome high-definition camera, connected to the PC via gigabit Ethernet and fitted with a 55 mm telecentric zoom lens (Computar). The camera was set to a frame rate of 33 fps and image resolution of 1280 × 960 pixels. A thin-walled glass tube (*Ø* = 1.5 cm) was used to contain cyprids in the field of view, when viewed from above, and was closed at both ends using glass coverslips. The tube was placed between the camera and a light source (either visible or infrared light-emitting diodes) for image acquisition (figure 2). It should be noted that while the materials described above were used in these specific experiments, an analogous system could be set up using any camera system with acceptable frame rate, contrast and available lenses, providing that in the captured movies the boundary of each cyprid includes at least five pixels.

To obtain cyprids, adult barnacles (*Balanus amphitrite*) were removed from seawater for 18 h and then returned to fresh seawater, where they released stage 1 nauplius larvae. A point light source was used to attract and collect the nauplii. The settlement-stage cyprid was reached in 4–5 days when nauplii were fed on an ad libitum diet of *Tetraselmis suecica* at 28°C. The cyprids were aged for 3 days at 6°C prior to use in experiments.

### 2.2. General structure of the tracking system

**Figure 3.**
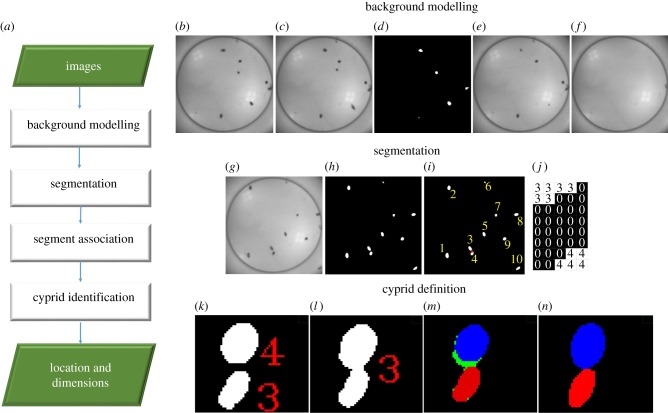
(*a*) Tracking system algorithm. (*b*) Initial background. (*c*) Next image. (*d*) Subtraction between the background and the next image. (*e*) Updated image. (*f*) Final background image. (*g*) An image from the tracking system, (*h*) binary image. (*i*) Labelled image. (*j*) Pixels in the third and fourth segments and background were set to be 3, 4 and 0, respectively. (*k*) Segments in previous image. (*l*) A segment in the current image. (*m*) Green is the free pixels. Red and blue are the remaining pixels. (*n*) Red and blue are the third and fourth cyprids, respectively.

Figure 3*a* shows a block diagram of the tracking system, in which images captured by a camera are processed through several steps to produce data describing the location, size and direction of the cyprids. A background subtraction method was chosen as the preferred means with which to isolate cyprids from the background for two reasons. First, a fixed camera was used and this maintained a constant, motionless background image enabling accurate background subtraction. Second, the illumination during the tracking procedure was relatively constant from start to finish. Had these conditions not been met, a more complex dynamic approach would have been necessary.

#### 2.2.1. Background modelling

Several techniques such as frame differencing, average filtering and mixture of Gaussians [32] can be used to define a background image. In this experimental design, the cyprid body was darker than the transparent surfaces on which it was exploring. Therefore, the background image could be easily extracted using the following steps:

(1) The first image was initially selected as a background image (figure 3*b*).
(2) The background image was subtracted from the next image (figure 3*c*).
(3) A threshold was applied (figure 3*d*).
(4) The corresponding bright pixels in the new image were copied into the background image (figure 3*e*).
(5) Steps 2–4 were repeated until no change to the background occurred (figure 3*f*).

#### 2.2.2. Segmentation

Cyprids in an image (figure 3*g*) were isolated by subtracting them from the background, applying a threshold (figure 3*h*) and then labelling the binary image. Each segment and its pixels were assigned a number as shown in figure 3*i*. Figure 3*j* was produced by zooming in the area of the red box in figure 3*i.* Pixels of the third segment were set as 3, and pixels of the fourth segment were set as 4, whereas the background pixels were set as 0. Segment size was calculated by counting the number of pixels contained within it, and the centre point of the body CE was determined by calculating the average value of its pixel coordinates using the following equation:2.1
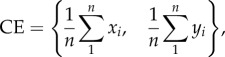
where *n* is the pixel number of the segment and (*x_i_*, *y_i_*) are the coordinates of the *i*th pixel.

#### 2.2.3. Segment association

Segments in the current and previous images were assigned as new and old segments, respectively. A new segment was associated with an old segment if they intersected each other by at least one pixel. In this case, the following situations were possible:

(1) One segment in the previous image remained one segment in the current image.
(2) More than one segment in the previous image touched or overlapped in the current image.
(3) A segment formed by more than one cyprid in the previous image was separated into many segments in the current image.
(4) A segment in the previous image moved outside of the camera's view in the current image.
(5) A new cyprid entered the camera view in the current image.

#### 2.2.4. Cyprid definition

Depending on the outcome of the segment association, the tracking algorithm may define cyprids in a number of different ways. The first case is that a new segment may be associated with no existing segment, or with one old segment carrying one cyprid. In this case, an ellipse-fitting algorithm described in [33] was used to define the cyprid. In the second case, a segment in the current image (figure 3*l*) may be associated with more than one segment (figure 3*k*). Here, two groups of pixels would be defined: the free and remaining pixels. The free pixel in the new segment (green colour in figure 3*m*) did not belong to any old segments. The remaining pixels (blue and red in figure 3*m*) were the overlapping pixels between the old and new segments. To separate the overlapped cyprids, the free pixels were added to the remaining pixels for each cyprid and an ellipse detection method was applied [34] (figure 3*n*).

After this, the following cost function was used to define the optimal ellipse to represent the cyprid:2.2
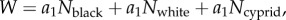
where *W* is the cost function value, *a*_1_, *a*_2_ and *a*_3_ are constants, and *N*_black_, *N*_white_ and *N*_cyprid_ are the number of the black pixels inside the ellipse, the number of the white pixels outside the ellipse and the number of the remaining pixels inside the ellipse.

The third possible case is that an old segment carrying more than one cyprid could be associated with more than one new segment. Each cyprid must therefore be assigned to the correct segment. To overcome this problem, the following cost function was applied:2.3
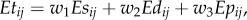
where *w*_1_, *w*_2_ and *w*_3_ are constants, *Et_ij_* is the total error, *Es_ij_*, *Ed_ij_* and *Ep_ij_* are the errors in the size, direction and the shared pixels between the cyprid *i* and the segment *j*. These three error terms are given by2.4
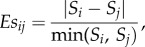
2.5
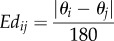
2.6
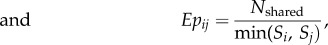
where *s* and *θ* are the size and direction, respectively. *N*_shared_ is the shared pixel between the cyprid *i* and segment *j*.

Finally, cyprids in old segments which are not then associated with any new segment were assigned as missed cyprids.

## 3. Development of a classification system for identifying cyprid behaviours

**Figure 4.**
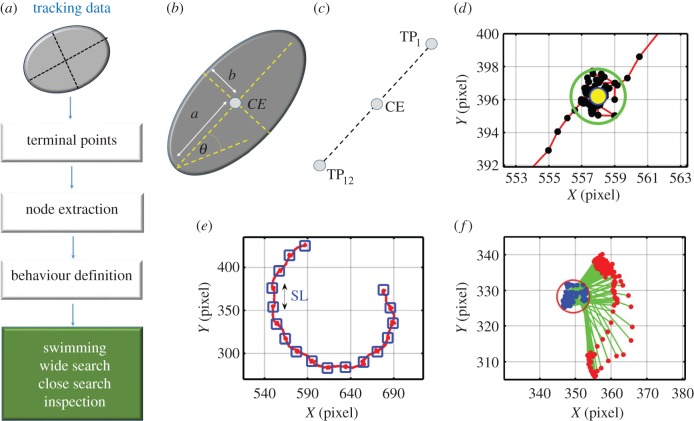
(*a*) Classification system. (*b*) A cyprid represented by an ellipse in the tracking system. (*c*) A cyprid represented by three points in the classification system. (*d*) A node extracted during walking behaviour. The red line represents the path of the cyprid centre point, the green circle is the boundary of the node, the yellow circle is the node centre and the small black points are locations of the cyprid centre recorded around the node. The radius of the node is 1.7 pixels, and the minimum number of node members in this case was 14. (*e*) Nodes extracted during the walking behaviour. SL is the step length. (*f*) An inspection event. The green lines represent the major axis of the ellipse. The endpoint TP_2_ formed a node, whereas TP_1_ oscillated about the node formed by TP_2_.

The block diagram in figure 4*a* is a schematic illustration of the classification system used to process data output from the aforementioned tracking system. Tracking data input was followed by three processing steps, namely identification of terminal points, node extraction and definition of behaviours. Following this process, tracking data were classified into one of four categories: swimming, wide search, close search or inspection behaviour.

### 3.1. Assigning terminal points

Prior to classification, the tracking system replaced each cyprid with an ellipse that was determined using four parameters: the centre point CE(*xc*, *yc*), semimajor *a*, semiminor *b* and the angle *θ* (figure 4*b*). The terminal points function block replaced each ellipse (cyprid) with three points; the centre and two terminal points (figure 4*c*). The terminal points (TP_1_(*tx*_1_, *ty*_1_) and TP_2_(*tx*_2_, *ty*_2_)) were located at the two ends of the major axis of the ellipse and were calculated using the following equations:3.1
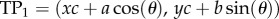
and3.2
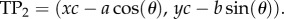


The three points for each cyprid were then processed though the next two block functions (figure 4*a*) to define the cyprid's movement.

### 3.2. Node extraction

For the purpose of classification, a node was defined as a set of points that met two conditions. First, the number of points must be above a threshold (*n*), and second, they must occur within a predefined radius. Figure 4*d* shows a node extracted during wide search behaviour, indicative of the pause taken by cyprids between exploratory steps. The node extraction function block thus extracted nodes from the three paths produced by the cyprid relating to the centre point and the two terminal points. The output from this function is then fed into the final stage of the classification system.

**Figure 5.**
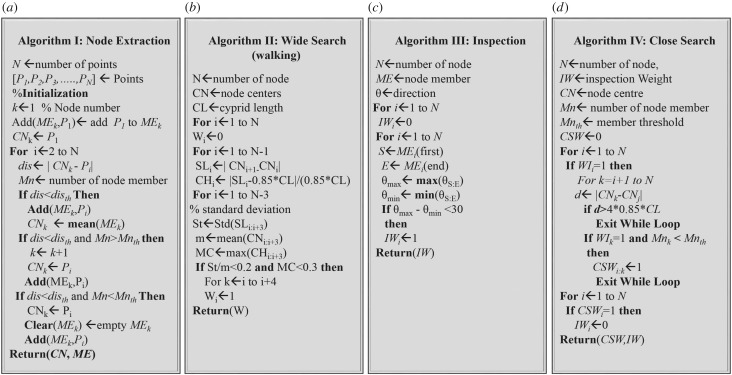
A summary of the classification algorithms. (*a*) The node extraction algorithm. (*b*) The wide searching classification algorithm. (*c*) Inspection classification algorithm. (*d*) The close search classification algorithm.

Figure 5*a* illustrates the node extraction algorithm, which applies the following steps to a set of points [*P*_1_, *P*_2_, *P*_3_, … ,*P_n_*] to find node centres CN and their members ME:

(1) The first node CN*_k_* is initiated with the first point *P_i_*_= 1_ and the index of this point (*i* = 1) is added to the node member array ME_*k* = 1_.
(2) A loop starts from *i* = 2 to *N* and the distance dis between *P_i_* and CN*_k_* is calculated and tested under three conditions:
  (a) The dis is smaller than the threshold distance dis_th_. This means the *P_i_* is located inside the node boundary. Therefore, the point index *i* is added to ME*_k_* and the CN*_k_* is recalculated by taking the average location of its members.
  (b) The dis is larger than dis_th_ and the number *Mn* of members in ME*_k_* is larger than the threshold *Mn*_th_. This means the *P_i_* is located outside the node boundary and the member points already stored in ME*_j_* achieve the two required conditions to create a node. Therefore, the node index *k* is increased by 1 to save the current node and to find a new node which is initialled with the *P_i_* and *i*.
  (c) In the third condition, the required two conditions were not met. Here, the CN*_k_* is reassigned with current point *P_i_* and the ME*_k_* is emptied and then reinitiated with the index *i*.

### 3.3. Definition of behaviours

The data produced during node extraction were used to empirically define the four behaviours under investigation. The observational component of this process was significant, requiring many hours of classification, error estimation, threshold adjustment and re-classification before the rates of error were diminished to a negligible level. The location of nodes in the three paths (centre and two terminal points) defined by cyprids during movement were thus used to classify behaviour as follows.

#### 3.3.1. Wide search

Wide search behaviour was identified using the nodes of the centre point (figure 4*e*). The path of an exploring cyprid is rarely a straight line, hence, assigning a wide search designation whenever a cyprid moves in a straight line [2] would produce a high rate of false classification. From observation of cyprids engaged in this behaviour, it was identified that the step length SL during walking (the distance between two consecutive nodes) most often met the following two conditions:

(1) The ratio between the standard deviation *St* and the average value *m* of SL is less than a threshold (0.2).
(2) The maximum change MC between the step length and 0.85 of the cyprid length is less than a threshold (0.3), with MC calculated as follows:3.3
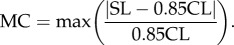

Stretches of movement in which the nodes fulfilled these two criteria were thus defined as wide search behaviour.

The algorithm for defining wide search behaviour is illustrated in figure 5*b.* The inputs of this algorithm are the nodes CN of the body centre and the cyprid length CL. The outcome is the weights *W* of nodes which take either of two values: ‘0’ for non-walking behaviour and ‘1’ for walking behaviour. In this algorithm, the following steps were implemented:

(1) The weights of all nodes [*W*_1_, *W*_2_, *W*_3_, … ,*W_n_*] were initiated with ‘0’.
(2) The distances (step lengths) SL*_i_* between two consecutive nodes [*P_i_*, *P_i_*_+1_] and the relative difference CH*_i_* between the SL*_i_* and 0.85 CL were calculated.
(3) A loop starts and *i* = 1 to *N* − 3 and carries out the following:
  (a) To calculate standard deviation *St* and mean *m* of the four consecutive step lengths [SL*_i_* to SL*_i_*_+3_].
  (b) To calculate the maximum change MC of four consecutive relatives [CH*_i_* to CH*_i_*_+3_].
  (c) To check if *St*/*m* and MC achieved the conditions required of walking behaviour, then covert the value of the weights [*W* to *W_i_*_+4_] to ‘1’.

#### 3.3.2. Inspection behaviour

During an inspection event, the cyprid will attach to the surface with its antennules and oscillate its body about that attachment point (figure 4*f*). This causes very little change in the location of the terminal point TP_2_, but a much larger movement at the opposite terminal point TP_1_. To automatically define an inspection event, it was found that the node of the attached terminal point must meet the following condition: the range of angles towards the various points TP_1_ with respect to the node must exceed a threshold of 30°.

Figure 5*c* illustrates the algorithm for detecting the inspection behaviour. The inputs of this algorithm are the number of nodes of a terminal point *N*, node member ME and cyprid directions *θ*. The output is the node weight IW. This algorithm implements the following steps:

(1) To initiate IW with zero values.
(2) To define the indexes of the first member *S* and last member *E* in the member array ME*_k_*.
(3) To find the maximum angle *θ*_max_ and minimum angle *θ*_min_ of the angles located between the *S* and *E.*
(4) To check if the inspection conditions are achieved and then to convert the node weight to 1.

#### 3.3.3. Close search

Close search behaviour was the most challenging to define and required a combination of the procedures used to identify wide search and inspection (hence they are described out of logical sequence). During this behaviour, the cyprids moved in a similar way to during wide search, but changed direction often, moving 1, 2, 3 or 4 steps along a trajectory before again changing direction. Because the classification system identified abrupt changes in direction anchored at a single point as inspection behaviour, it was possible to have close search behaviour defined as a series of inspection events (nodes) when the maximum distance between consecutive nodes was smaller than four steps (less than 4 × 0.85 CL)*.* However, cyprids occasionally moved from close search behaviour to inspection behaviour, which lasted several minutes, before returning to close search. Therefore, another condition was added to more clearly define close search behaviour, i.e. the number of the node must be less than a threshold *Mn*_th._

Figure 5*d* illustrates an algorithm for defining close search behaviour. The inputs of this algorithm are the inspection node weights IW, node centres CN and the length of the cyprid CL. The output is the close search weights CSW. This algorithm carries out the following steps:

(1) To initiate CSW with zero values.
(2) To check the IW weights one by one.
(3) If the current weight IW*_i_* is equal to ‘1’, check if the next node is located within a distance less than four steps, has a weight IW*_k_* equal to ‘1’ and its member is less than the number threshold (*Mn_k_* < *Mn*_th_), then [CSW*_i_* to CSW*_j_*] convert to ‘1’.
(4) To remove the overlap between inspection and close search behaviour, each corresponding inspection weight for a close search weight with value ‘1’ is converted to ‘0’.

#### 3.3.4. Swimming

The motion of the cyprid was classified as swimming when the previous three behaviours were not detected.

## 4. Examples of quantitative data output

Two basic experiments illustrate the ability of the tracking system to identify differences in the behaviour of cyprids under different experimental treatments and in a single treatment over an extended time period.

### 4.1. Comparison of lighting conditions

In experiment 1, two lighting conditions were used, namely illumination by infrared or visible light. Twenty-five cyprids were recorded simultaneously for 25 min under IR light in dark conditions. The IR source was then replaced by visible light, and the same cyprids were then captured for a further 25 min. Cyprids were allowed a 3 h acclimation period prior to the experiment to stabilize their behaviour, and the temperature of the experimental chamber was maintained at a constant 25°C.

Table 1 contains output from the classification system. Wide search behaviour was quantified by automatic counting of the number of steps per wide search event by the classification system. Inspection and close search events were quantified by duration. Wide search is the behaviour most commonly investigated with regard to surface selectivity by cyprids [31]. It is a characteristic cyprid behaviour, but comparatively rare on a per-cyprid basis when expressed as a proportion of total time spent moving. No wide search events were detected under IR light, whereas a single cyprid engaged in one stretch of wide search under visible light, carrying out a total of 13 steps. It would be impossible, therefore, to discriminate between conditions on the basis of wide search alone as has been attempted previously, and data for close search were also similar between treatments. One close search event was detected for each lighting condition. The close search event under IR light was longer in duration, measuring 101 s compared with 52 s under visible light.

**Table 1.** Results produced by the classification system.

	visible			IR		
	wide search	close search	inspection	wide search	close search	inspection
event number	1	1	11	0	1	2
total	13 (step)	52 (s)	1949 (s)	—	101 (s)	45 (s)
average	13 (step)	52 (s)	177 (s)	—	101 (s)	22 (s)

**Figure 6.**
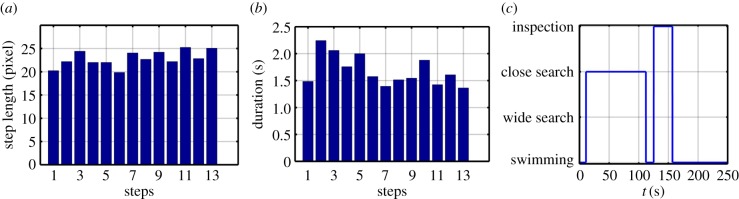
(*a*) The durations of the walking steps measured in frames. (*b*) The step lengths measured in pixels. (*c*) The sequence in the behaviour of cyprid 6.

The most notable difference between treatments was in the inspection behaviour of cyprids. The total number of events and average duration of the inspection events under visible light were markedly larger than those under IR. Eleven inspection events were detected under visible light with a total duration of 1949 s and average duration of 177 s. On the other hand, only two events occurred under IR light with a total duration of 45 s and average duration of 22 s. From the classification data, further useful information was extracted to better describe cyprid wide search behaviour, including the distance between steps (figure 6*a*), the duration of each step (figure 6*b*) and the exact time points of transitions between behaviours of interest. For example, cyprid number 6 (figure 6*c*) started close search behaviour at *t* = 11 s, finished at *t* = 112 s swam until *t* = 125 s and then started inspection behaviour which finished at *t* = 156 s. Behavioural monitoring with this degree of precision has never before been accomplished for barnacle larvae and stands to advance significantly our understanding of the process of surface selection.

### 4.2. Long-term tracking

**Figure 7.**
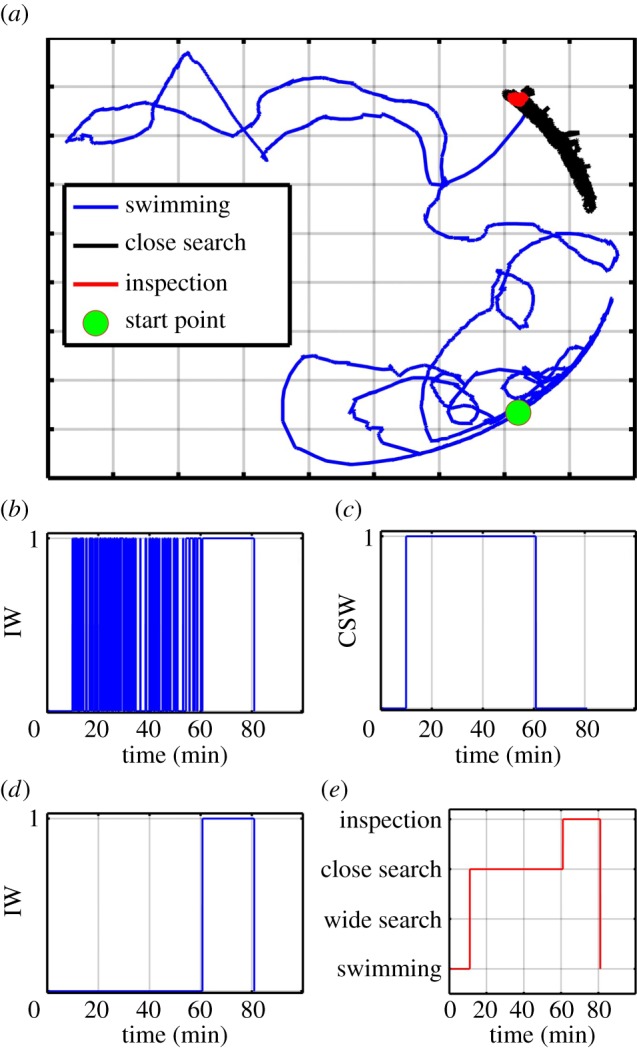
Data relating to a cyprid that settled during a long-term tracking experiment. (*a*) The cyprid trajectory leading up to settlement. (*b*) The outputs of applying the inspection classification algorithm. (*c*) The inspection weights after applying the close search algorithm. (*d*) The weights of the close search behaviour. (*e*) The overall behaviour of the cyprid.

A second experiment was performed to test the classification system. The aim of this experiment was to show that the classification system is able to identify a sequence of behaviour carried out up to and including permanent attachment of the cyprid to the surface. First, 25 cyprids were monitored and recorded by the image acquisition system over a period of 5 h, during which progress was regularly checked by a user but no intervention was required. After 5 h a cyprid settled and the image acquisition system was terminated. The tracking system was used to process the stored images and track the cyprid from the point of settlement backwards for 80 min, at which time the individual was swimming in the water column. Next, the classification system was applied to the data delivered by the tracking system. Surprisingly, during 80 min of surface-specific behaviour, the cyprid under investigation did not engage in any wide search behaviour, according to the definitions described above. This was confirmed by the user. The inspection classification algorithm produced the inspection weights IW shown in figure 7*a*, from which events of inspection can be seen. As mentioned before, close search behaviour is detected if many separate inspection events occur in a small area. Therefore, to detect close search, the inspection weights IW were fed into the close search classification algorithm, which modified the inspection weights and extracted the close search behaviour data as displayed in figure 7*b*,*c*, respectively. The overall behaviour of the cyprid was described by combining the weights of the inspection and close search behaviours as shown in figure 7*d*. After an arbitrary 11 min of swimming the cyprid began its surface exploration behaviour, engaging in close search behaviour for 50 min and then inspection behaviour for 19 min before attaching and metamorphosing. The cyprid trajectory during 80 min is displayed in figure 7*e* where the blue, black and red colours represent the trajectory of the cyprid during the swimming, close search and inspection behaviour, respectively. The system thus demonstrated its ability to record, track and classify cyprid behaviours precisely and with the minimum of error for an extended period of time, providing a new experimental capability that will significantly accelerate our understanding of cyprid pre-settlement behaviour and the relation of this to surface selection.

## 5. Discussion

Since the experiments of Crisp [10], who used a camera lucida to trace the tracks of exploring cyprids, the same barriers have remained to furthering our understanding of surface selection by barnacle cyprids, even with the advent of sophisticated two- [26] and three-dimensional [28] video-tracking techniques. The exploratory phase of cyprid settlement behaviour in the laboratory can be long—Visscher [35] referred to over an hour for *Balanus* spp.—and punctuated by frequent periods of swimming, and the subtleties of the behaviours are probably important yet difficult to differentiate computationally. All approaches proposed so far have been labour intensive and required, at some stage, the opinion of a human experimenter to interpret the data, mitigating any benefit of an automated tracking method. On the contrary, the tracking and classification systems described here represent the first successful implementation of software to record and track multiple cyprids simultaneously for the durations necessary to capture the complete suite of behaviours leading to settlement. The data produced are piped to a bespoke classification system that automatically and reliably differentiates and quantifies the standard cyprid behaviours of swimming, wide search, close search and inspection, as defined by Crisp [10]. Elimination of the experimenter from the tracking process, except for periodic observation for quality control, provides true standardization between experiments, and the single camera system produces a relatively low data burden, allowing for tracking experiments of many hours in duration.

Automatic management of overlapping events is a major step forward in this process, permitting long-term monitoring of individual cyprids and, for the first time, precise description of the behaviours leading to settlement for multiple larvae. This was achieved using an ellipse-fitting algorithm, which analyses non-overlapping pixels of the cyprids and new pixels appearing in subsequent frames, to find the optimal shape. The problem of defining cyprids when they separate was solved using three parameters, namely their shared pixels, the error in their sizes and the error in their directions. For classification of behaviours, cyprids were treated as three points—the centre point and two terminal points. The distances between nodes extracted from the locations of the centre point were processed to define wide exploration and nodes extracted from the two terminal points were used to define inspection events, which were then processed again to detect close search behaviour.

Comparison of the outputs from the classification system with the observations of an expert user showed that the classification system made remarkably few errors. Furthermore, the classification system was able to precisely define the sequence of behaviours exhibited by a cyprid in the hours before settlement. These advances pave the way for future experiments that will, in the first instance, produce robust baseline data for the process of exploration and settlement in model fouling barnacle species, to identify ‘normal’ behaviour. With this knowledge, it will then be possible to examine numerous fundamental and applied aspects of cyprid behaviour including the response to conspecifics, biofilm-conditioned surfaces and developmental fouling-resistant coatings. With regard to the latter, a clear application for this technique is comparison during laboratory evaluation of coatings that cyprids reject during standard settlement assays [26].

As for all behavioural analysis, the usefulness of the output is determined by the parametrization of the data in the context of the behaviours of interest. While the system described here solves the technical issues of duration, resolution and complexity of cyprid-tracking experiments, it creates its own challenges. Previous behavioural analysis systems have relied upon the precise acquisition of coordinate data for reconstruction of tracks and later interpretation. Our system, on the other hand, recognizes behaviour directly and all measurements are relative. This circumvents the need for laborious calibration procedures and provides the experimenter with the information they require directly. Nevertheless, spatially calibrated measurements of, for example, step length and walking speed are not provided, but these can be derived from the more traditional behavioural analysis methods discussed already.

In comparison with the data we are now able to acquire, the ‘Crisp model’ of cyprid exploratory behaviour can be considered ‘low resolution’. It was developed from a collection of short exploratory tracks from cyprids of indeterminate age and ‘experience’, and the model is thus applicable only for providing general context for cyprid behaviours within an average exploration/settlement sequence. The data produced by the method described here are individual-specific, high magnification, long term and therefore of higher resolution. Precise durations and sequences are available for particular cyprids in the lead up to settlement and it is therefore possible that, as data amass, they indicate necessary refinements to the Crisp model. For example, the cyprid tracked in figure 7 never engaged in wide searching between removal from the larval culture and settlement in the experimental chamber. This example and the observations of barnacle researchers elsewhere [36] indicate that shortcuts through the model are certainly possible, and may be more common in some conditions than others.

The definition of close search behaviour was particularly challenging in the face of increased data resolution, and may yet require modification. Crisp's description of this behaviour was that larvae turn more frequently than in wide searching and that the behaviour generally occurs between wide searching and inspection. In reality, a track conforming to this definition can arise in a number of different ways and these probably indicate different degrees of stimulation of the larva, or larval physiological state [37]. For example, there is continuous, tortuous, surface exploration which is unequivocally close search, according to Crisp's definition. Second, cyprids engaged in long periods of linear walking may occasionally make changes in direction in a restricted area before beginning again on a linear path. Or, third, short stretches of linear walking (identified by the classification system as wide searching) may be interspersed by inspection events, with the frequency of inspection events per exploratory step varying and, presumably, containing useful information. Depending on the thresholds of observation, the latter may be identified as either alternating wide search or inspection events, or as close search. At low resolution, all of the above paths would appear to be close search, but the means by which they are produced, and probably their meanings, are very different. Simply eschewing low-resolution observation in preference for more detailed data, therefore, is not a simple solution. In this case, a logical progression from the close search example is to ask whether the existing definition of inspection is truly representative (because at high resolution close search may comprise periods of inspection and wide search). Crisp described inspection as occurring immediately before permanent attachment. In which if the same behaviour occurs at other points in a prolonged period of surface exploration, should it still be referred to as inspection? Or is such detail unhelpful and, instead, a more global view should be taken, looking instead at ‘average’ behaviour over time?

As the research progresses it is possible, even likely, that the definitions of Crisp will be inadequate to describe the nuances of cyprid exploratory behaviour. Indeed, it may be that classification of exploratory behaviour with the purpose of identifying predictive patterns is, in itself, an overly simplistic approach and that more sophisticated machine learning, artificial intelligence and pattern recognition techniques are more appropriate [38]. In any case, the method described herein provides a straightforward and accessible way to gather reliable high-resolution data that can subsequently be fed into models of cyprid behaviour. This, in itself, is a significant step forward.
